# The Evaluation of Memantine Effect on Tinnitus Severity

**DOI:** 10.1002/brb3.70697

**Published:** 2025-07-20

**Authors:** Babak Pourayyoubi, Alireza Rezaei‐Ashtiani, Javad Javaheri, Mohsen Ebrahimi Monfared, Farzad Zamani

**Affiliations:** ^1^ Student Research Committee, School of Medicine Arak University of Medical Sciences Arak Iran; ^2^ Department of Neurology, School of Medicine Arak University of Medical Sciences Arak Iran; ^3^ Department of Community Medicine, School of Medicine Arak University of Medical Sciences Arak Iran; ^4^ Department of Otorhinolaryngology, School of Medicine Arak University of Medical Sciences Arak Iran

**Keywords:** memantine, NMDA receptors, tinnitus

## Abstract

**Purpose:**

Tinnitus is a common condition where a person perceives sound despite there being no external auditory stimuli. It is proven that overexpression of NMDA (*N*‐methyl‐d‐aspartate) glutamate receptors increases the sensitivity of neurons to glutamate transmission, creating a destructive cycle of excitotoxicity. NMDA receptor antagonists, such as memantine, seem to be effective in treating tinnitus. This study aimed to examine the effectiveness of memantine as a treatment for tinnitus in a double‐blind randomized placebo control clinical trial.

**Methods:**

The participants were patients with tinnitus. A total of 70 patients were randomly assigned into two groups, intervention and a placebo, with an equal number of patients in each group. Both groups received conventional treatment with cinnarizine at a dose of 25 mg twice a day. In the intervention group, memantine was added to cinnarizine with starting dose of 5 mg. Tinnitus Severity Index (TSI) and Numeric Rating Scale (NRS) were recorded before and after study in both groups.

**Findings:**

There was no significant difference in the initial mean of NRS and TSI scores between two groups. After treatment, the mean NRS and TSI scores were significantly lower in the intervention group. Although the changes in mean NRS scores were significant in both groups at the end of study but only the mean TSI scores were significantly decreased in the intervention group.

**Conclusion:**

On the basis of these findings, it is suggested that memantine may have remarkable effect in reducing tinnitus and its discomfort.

## Graphical abstract

The graphical abstract illustrates the study structure: 70 tinnitus patients were randomly assigned to two groups for a 60‐day treatment. The image shows excitotoxic mechanisms, intervention, and outcomes in a concept‐based layout.

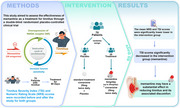

## 1 Introduction

Tinnitus refers to the perception of sound or noise in the absence of any external or internal acoustic stimulation. This condition affects approximately 10%–15% of the general population, with severe tinnitus impacting around 1%–2% of individuals, causing distress and interfering with normal life activities (Hoffman and Reed 2004). This complication can seriously impair the ability to sleep and rest and cause fatigue, nervousness, irritation, and depression (Moghtaderi et al. 2012). Effective treatment options are limited, and the mechanisms underlying the generation and maintenance of tinnitus are not yet fully understood.

Research has focused on the glutamatergic system as a potential contributor to tinnitus, as glutamate is the main excitatory neurotransmitter in both the cochlea and central auditory pathways. The overexpression of NMDA (*N*‐methyl‐d‐aspartate) glutamate receptors increases the sensitivity of neurons to glutamate transmission and keeps the vicious cycle of destruction caused by excitotoxicity (Zheng et al. 2006; Denk et al. 1997; Oestreicher et al. 1998). Evidence has shown that during this critical process, auditory nerve dendrites may be particularly susceptible to some stimulation by the NMDA receptor (NR) and thus produce an imaginary sound called tinnitus (Oestreicher et al. 2002). Excessive release of glutamate has been hypothesized to lead to overexpression of NMDA synaptic receptors, which may cause swelling and rupture of neurons due to excessive entry of calcium and water (Pujol et al. 1993; Guitton et al. 2003).

Studies have shown that functional changes in the cochlea and central nervous system (CNS) are involved in the pathophysiology of tinnitus, and neuroplastic changes in the CNS are largely dependent on NMDA‐mediated neurotransmission. Upregulation of glutamate receptors has been observed in the cochlear nucleus of animal models of tinnitus (Pujol et al. 1993; Weisz et al. 2007).

In summary, tinnitus remains a challenging condition to treat, and the mechanisms underlying its generation and maintenance are not fully understood. The glutamatergic system has been proposed as a potential contributor to the pathophysiology of tinnitus. Further research is needed to fully elucidate the mechanisms underlying tinnitus and to develop effective treatment options.

Therefore, drugs that act as NR antagonists are thought to be effective in treating tinnitus. Memantine is one such drug.

Memantine is a type of medication that works as a noncompetitive antagonist of NR. It helps reduce the release of excessive levels of glutamate, which can cause overstimulation of neurons (Hwang et al. 2011). However, there have been only a few published studies on the effect of memantine on tinnitus, and the results are conflicting. Additionally, the studies that do exist have only focused on the behavioral symptoms associated with memantine.

Several studies have been conducted to investigate the effects of different drugs on tinnitus in both humans and animals. A double‐blind clinical trial was conducted in the United States by Ricardo Rodrigues Figueiredo et al. to investigate the effect of memantine on tinnitus. However, the examination results did not show any significant difference between the group that received the drug and the placebo group. The study concluded that the effect of memantine on tinnitus was not proven (Figueiredo et al. 2008). Some studies have been conducted to investigate the effect of memantine on the salicylate‐induced tinnitus in rats. The first animal study was conducted in Italy by Ralli et al. (2014) that found memantine significantly reduces tinnitus‐like complications in mouse models, although the location of the memantine effect was not determined. The second study was conducted by Chul et al., the effects of memantine on a salicylate‐induced tinnitus model were investigated. The researchers first induced tinnitus in rats by administering salicylate and then evaluated the effects of memantine on the rats. The results showed that memantine was able to significantly reduce the severity of tinnitus in the rats. The drug was found to work by blocking a type of receptor in the brain that is responsible for the development of tinnitus (Jang et al. 2019).

In another study in New Zealand by Zheng Y, et al., the effect of memantine on chronic tinnitus caused by noise trauma was examined. The conclusion was that memantine has a positive impact on improving tinnitus caused by vocal injury (Zheng et al. 2012).

On the basis of a limited number of studies on the effect of memantine on the severity of tinnitus in humans, we have decided to conduct a randomized, double‐blind placebo controlled study to investigate the effectiveness of glutamate receptor blockers, specifically memantine, in treating tinnitus in humans.

## 2 Materials and Methods

### 2.1 Study Design

This study was a randomized, double‐blind, placebo‐controlled clinical trial conducted to evaluate the efficacy of memantine, in combination with cinnarizine, on tinnitus severity. The trial was carried out at the Neurology Clinic of Vali‐Asr Hospital and the ENT Clinic of Imam Reza Polyclinic, both affiliated with Arak University of Medical Sciences (AUMS), Iran.

### 2.2 Participants and Eligibility Criteria

A total of 94 individuals with tinnitus complaints were assessed for eligibility. Of these, 24 participants were excluded due to not meeting the inclusion criteria or declining to participate. Ultimately, 70 eligible participants were enrolled and randomly assigned to two equal groups (*n* = 35 per group).

**Inclusion criteria** were as follows: age between 18 and 65 years, both sexes, and provision of written informed consent.

**Exclusion criteria** included middle or inner ear diseases, temporomandibular joint disorders, use of drugs affecting the central vestibular system in the past 6 months, tinnitus duration less than 2 months, thyroid disease, rheumatologic conditions, uncontrolled hypertension, renal failure, moderate to severe anemia, space‐occupying brain lesions, irregular medication adherence, lack of follow‐up access, serious hearing impairment, or intolerance to the study drugs.

### 2.3 Randomization and Blinding

Participants were randomized using a random number table by a research advisor not involved in clinical assessments. A sequential method was employed in which the first eligible participant was randomly assigned to one group, followed by the second to the alternate group. Randomization ensured balanced distribution of potential confounding factors between the two groups.

Blinding was maintained at a double‐blind level: Neither the participants nor the examining physicians were aware of treatment allocation. Identical‐appearing packages containing memantine or placebo were coded and distributed. Dose titration instructions were standardized for both groups to preserve masking.

### 2.4 Intervention

All eligible patients underwent vital sign checks, audiometric tests, and blood tests (including complete blood count, fasting blood sugar, creatinine, and blood urea) to record their baseline information such as age, sex, education, residency, occupation, and symptoms like dizziness and feeling of ear fullness. For analytical purposes, “accompanying symptoms” were defined as additional subjective complaints reported alongside tinnitus, including dizziness, a sense of ear fullness, and sleep disturbances.

All participants received cinnarizine 25 mg twice daily as part of the standard baseline treatment. This decision was based on empirical usage in Iranian clinical settings and ethical recommendations to avoid withholding a commonly prescribed medication.

The intervention group received memantine in an escalating dose:

- 5 mg/day for the first 2 weeks;
- 10 mg/day in weeks 3–4;
- If no improvement was reported, the dose was increased to 15 mg in weeks 5–6;
- Finally, if necessary, increased to 20 mg/day for the last 2 weeks.

The placebo group received a visually identical placebo under the same protocol. No cognitive behavioral therapy or tinnitus retraining therapy was administered to any participant during the study period.

### 2.5 Dropout and Replacement

During the study, nine participants were lost to follow‐up, four in the memantine group (due to mild gastrointestinal side effects, loss of hope for improvement, and unreachability) and five in the placebo group (due to similar reasons). To maintain the predetermined sample size, these participants were replaced with new eligible individuals meeting the same criteria. Ultimately, 35 participants in each group completed the study and were included in the final analysis. A CONSORT flow diagram is provided to illustrate the participant enrollment, allocation, dropout, replacement, and final analysis process.

### 2.6 Outcome Measures

Tinnitus severity was evaluated using two validated tools at baseline and after 60 days:

- **Tinnitus Severity Index (TSI)**: A 12–60 point scale assessing the impact of tinnitus. Severity levels were categorized as follows: very mild (1–12), mild (13–24), moderate (25–36), severe (37–48), and catastrophic (49–60).
- **Numeric Rating Scale (NRS)**: A 0–10 point scale evaluating perceived loudness and distress due to tinnitus, where 0 indicates no tinnitus and 10 denotes maximum severity.

### 2.7 Audiometric Evaluation

All participants underwent pure tone audiometry using a Madsen Orbiter 922 clinical audiometer and TDH‐39 headphones. Thresholds were recorded at frequencies of 250, 500, 1000, 2000, 4000, and 8000 Hz. A pure tone average <25 dB HL was considered indicative of normal hearing.

### 2.8 Adverse Events Monitoring

Participants were monitored throughout the study for adverse events via clinical follow‐up and self‐reporting. Mild side effects such as nausea or gastrointestinal discomfort were reported only in the memantine group. These resolved spontaneously within approximately 10 days, facilitated by gradual dose titration and administration after meals.

**Ethical approval** for this study was obtained from the Medical Research Ethics Committee of AUMS, under code number 93.174.19. The authors affirm that all procedures involved in this research adhere to the ethical standards set forth by relevant national and institutional guidelines, in accordance with the Helsinki Declaration of 1975, as revised in 2008.

### 2.9 Statistical Method of Data Analysis

All statistical analyses were performed using IBM SPSS Statistics version 28 software (IBM Corp., Armonk, NY; RRID:SCR_019096). Data were expressed as mean ± standard deviation (SD) for continuous variables and as frequency (percentage) for categorical variables. The normality of data distribution was assessed using the Kolmogorov–Smirnov test. The independent *t*‐test was used to analyze quantitative variables, whereas chi‐square (*χ* ^2^) was used for qualitative variables. The Mann–Whitney test was used to compare the data with abnormal distribution. To control for potential confounding variables and assess the independent effect of the intervention on the outcome, a multivariate linear regression analysis was performed. Variables with a *p* value <0.1 in univariate analysis were entered into the multivariate model. Statistical significance was set at *p* < 0.05. Data analysis was performed on participants who completed the study, following a per‐protocol approach.

## 3 Results

Seventy participants were randomized (35 per group), and all completed the study after replacing dropouts. The study was included 70 patients with a mean age of 50.14 (±9.14) years. Of these patients, 34 (48.57%) were male, and 36 (51.43%) were female. Table 1 provides baseline demographic and epidemiological information about the patients, for which there was no statistical significant difference between two groups.

**TABLE 1:** Demographic, epidemiological, and clinical information of patients in the intervention (M) and control (P) groups.

Parameter		M group	P group	*p* value
Age (mean/SD)		49.91/9.93	50.37/8.43	0.42
Duration of tinnitus (mean month)		8.7	9.0	0.58
Nature of tinnitus (%)	Tonal	60	57/14	0.73
	Noise	40	42/85	
Gender (%)	Male	54.28	48.57	0.632
	Female	45.71	51.42	
Education (%)	Uneducated	28.57	14.28	
	High‐school	28.57	14.28	
	Diploma	25.71	45.71	0.322
	Bachelor	14.28	14.28	
	Master or high	02.85	00.00	
Residency (%)	Urban	71.42	62.85	0.360
	Rural	28.57	37.14	
Job (%)	Un‐employed	37.14	34.28	0.079
	Employed	62.90	65.72	
Experiencing additional symptoms alongside the main ones (%)		40.00	65.70	0.055
Dose (%)	10 mg	25.74	—	
	15 mg	45.71	—	—
	20 mg	28.57	—	

*Note*: Analysis based on participants who completed the study (*n* = 35 per group).

During the course of the study, a total of nine participants were lost to follow‐up: four from the memantine group and five from the placebo group. Reasons for dropout in the memantine group included one case of gastrointestinal side effects, one due to lack of hope for improvement, and two who became unreachable. In the placebo group, three participants discontinued due to lack of hope for improvement, and two became unreachable.

**DIAGRAM 1:**
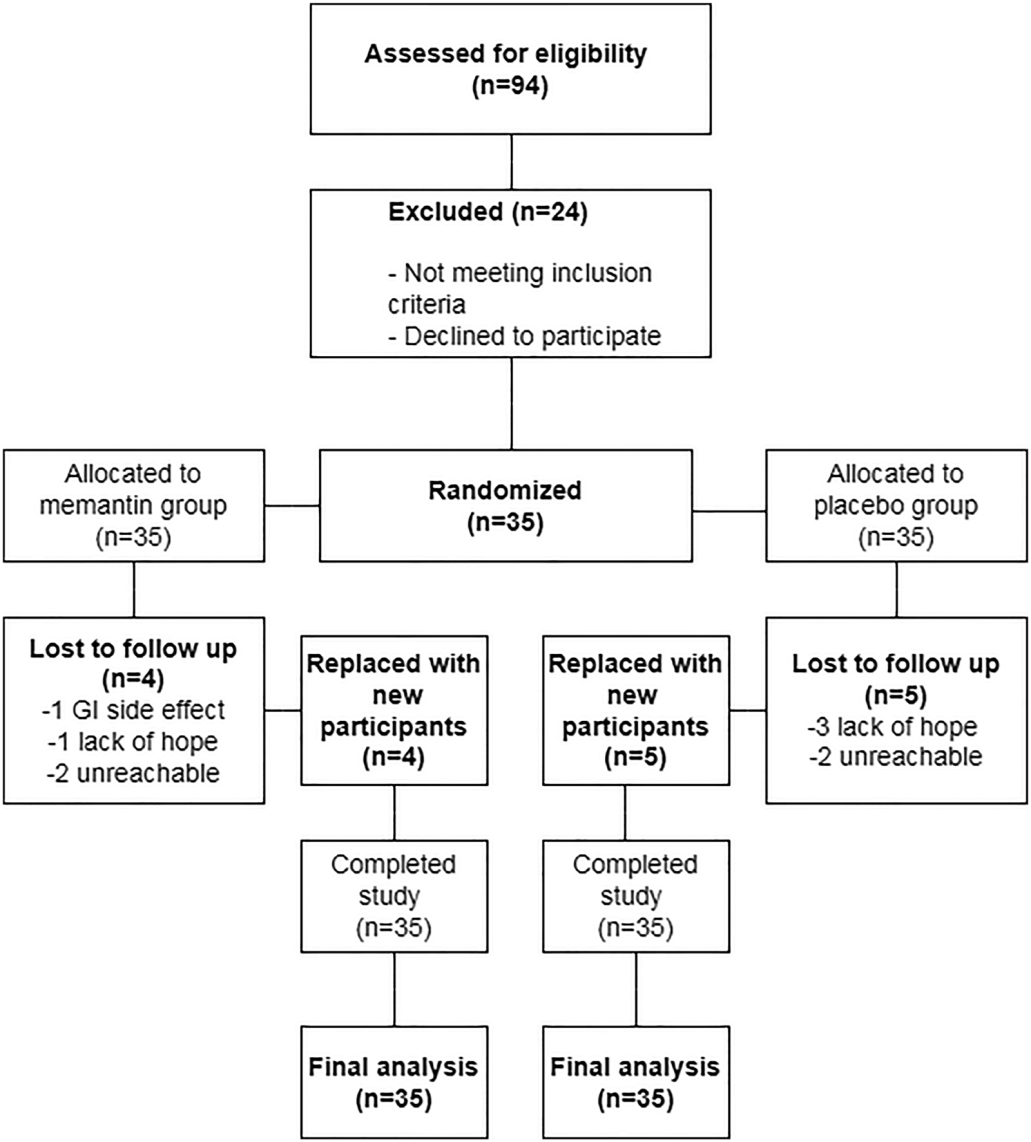
**CONSORT flow diagram** illustrating the enrollment, allocation, follow‐up, and analysis of participants in the study.

To maintain the original sample size, these dropouts were replaced with new participants, according to the inclusion criteria. As a result, both groups retained 35 participants each who completed the study and were included in the final analysis (Diagram 1).

The results of the pure tone audiometry test (Table 2) indicate that the participants generally have auditory thresholds within the normal to mild hearing loss range. Specifically, at lower frequencies (such as 250–1000 Hz), the average thresholds are close to normal hearing levels. At higher frequencies (2000–8000 Hz), there is a slight increase in thresholds; however, this increase is not significant enough to suggest deafness or severe hearing loss. Additionally, the *p* values provided (which are all above 0.05 for each frequency) demonstrate that there is no statistically significant difference between the two groups. Therefore, it can be concluded that the participants in both groups have similar hearing ranges.

**TABLE 2:** The results of the pure tone audiometry test

Frequency (Hz)	M group	P group	*p* value
250	12.3 ± 3.2	12.1 ± 3.4	0.82
500	16.8 ± 2.8	16.9 ± 2.9	0.91
1000	18.1 ± 3.2	18.0 ± 3.3	0.95
2000	22.4 ± 3.6	22.2 ± 3.5	0.88
4000	28.5 ± 4.0	28.8 ± 4.0	0.78
8000	33.7 ± 4.2	33.5 ± 4.3	0.85

*Note*: Analysis based on participants who completed the study (*n* = 35 per group). Records are indicated as mean dB ± SD.

The information regarding the mean NRS standard in the intervention and control groups can be found in Table 3. According to the results, there was no significant difference between the mean NRS before treatment of the intervention (7.68) and control (8.11) groups, with a *p* value of 0.130.

**TABLE 3:** Mean ± standard deviation (SD) numeric rating scale (NRS) criterion before and after the treatment in intervention and control groups.

Parameter	M group	P group	*p* value
NRS (before the treatment)	7.68 **± **1.13	8.11 **± **1.36	0.130
NRS (after the treatment)	3.17 **± **1.72	6.91 **± **2.94	<0.001

*Note*: Analysis based on participants who completed the study (*n* = 35 per group).

However, after the treatment completion, the mean NRS of the intervention group (3.17) was significantly lower than that of the placebo group (6.91), with a *p* value of <0.001.

**FIGURE 1:**
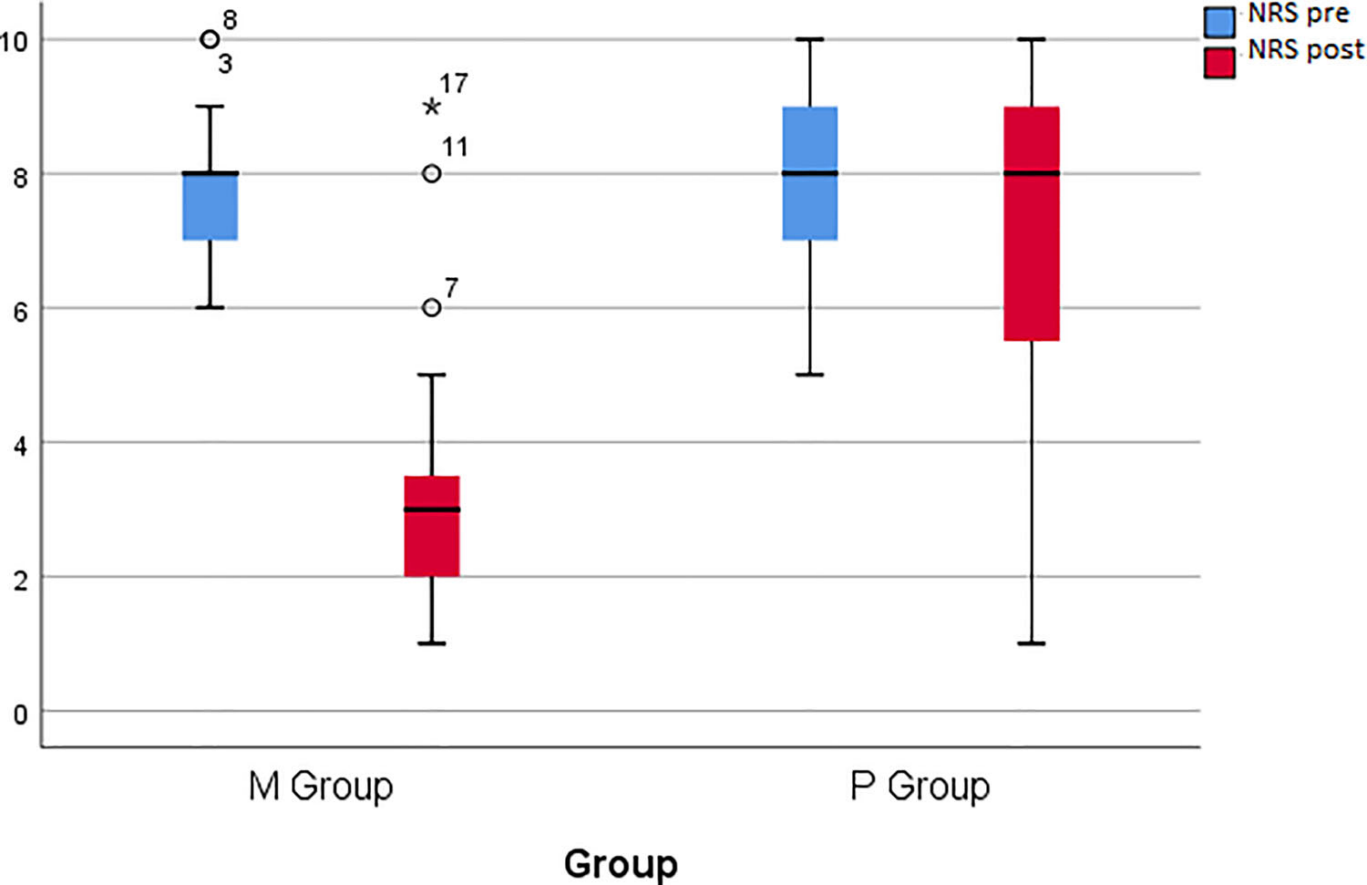
Changes in the mean NRS during 60 days of treatment in the intervention and control groups. NRS, Numeric Rating Scale.

The following text presents the changes in NRS during 60 days of treatment in both the intervention and control groups, as illustrated in Figure 1. The results revealed that the mean NRS significantly decreased in both the intervention (*p *< 0.001) and control (*p* = 0.01) groups during treatment.

Table 4 provides information on the mean TSI criterion in the intervention and control groups. The results show that there was no significant difference between the mean TSI before treatment in the intervention (46.45) and control (47.34) groups (*p* = 0.433). However, after treatment, the mean TSI in the intervention group (18.91) was significantly lower than that of the control group (45.71) (*p* < 0.001).

**TABLE 4:** Mean ± standard deviation (SD) Tinnitus Severity Index (TSI) criterion before and after the treatment in intervention and control groups.

Parameter	M group	P group	*p* value
TSI (before the treatment)	46.45 **± **4.87	47.34 **± **4.48	0.433
TSI (after the treatment)	18.91 **± **8.08	45.71 **± **5.02	<0.001

*Note*: Analysis based on participants who completed the study (*n* = 35 per group).

**FIGURE 2:**
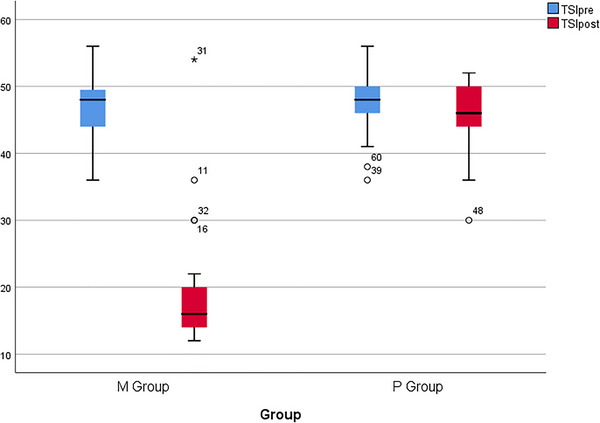
Changes in the mean TSI during the 60 days of treatment in intervention and control groups. TSI, Tinnitus Severity Index.

The changes in average TSI during 60 days of treatment in both the intervention and control groups are displayed in Figure 2. The findings revealed that the average TSI in the intervention group decreased significantly (*p* < 0.001), whereas there were no significant changes observed in the control group (*p* = 0.05).

To evaluate the effect of memantine and other potential predictor variables on tinnitus severity, a multiple linear regression analysis was performed using the enter method. The model showed a statistically significant goodness of fit (ANOVA *p* < 0.001), with an adjusted *R* ^2^ of 0.808, indicating that approximately 80.8% of the variance in tinnitus severity could be explained by the included variables (Table 5).

**TABLE 5:** Results of multiple linear regression analysis predicting final Tinnitus Severity Index (TSI) score based on clinical and demographic variables.

Predictor variable	*B* (unstandardized coefficient)	*p* value
Group (memantine)	34.231	<0.001
Dose	0.513	0.172
Accompanying symptoms	−1.651	0.356
Age	−0.011	0.912
Sex	1.325	0.587
Education	−1.272	0.191
Residency	0.291	0.882
Job	2.827	0.057
Duration of tinnitus	1.207	0.208
Nature of tinnitus	2.304	0.255

*Note*: Analysis based on participants who completed the study (*n* = 35 per group). Model summary: adjusted *R* ^2^ = 0.808. ANOVA (*F*‐test): *p* = 0.000. *p < 0.05 is considered statistically significant*.

Among the predictors, only the treatment group (memantine) was found to be a significant predictor of tinnitus severity (B = 34.231, *p* < 0.001). Other variables, including dose, accompanying symptoms, age, sex, education, residency, occupation, duration, and nature of tinnitus, did not significantly predict tinnitus severity (all *p* > 0.05). These findings suggest that memantine had a significant independent effect on tinnitus reduction after adjusting for other predictor variables.

## 4 Discussion

Our study results strongly support the theory that NMDA glutamate receptors play a critical role in the development of tinnitus. This occurs through a vicious cycle of excitotoxicity. As a result, memantine emerges as a promising intervention for effectively reducing the severity of tinnitus symptoms and related discomforts. Chul et al. recently conducted a study to examine the impact of memantine on salicylate‐induced tinnitus and concluded that overexpression of the NR2B gene of NMDA glutamate receptors significantly contributes to the development and severity of tinnitus (Jang et al. 2019). Our study was able to validate these findings. Despite being conducted on rats, the results of both studies were nearly identical, highlighting a strong association between glutamate and the overexpression of NRs in the development of tinnitus. Furthermore, the use of antagonists such as memantine can control and alleviate the severity of tinnitus and the associated discomfort. These findings align with another study conducted by Ralli et al. (2014), which concluded that the combination of memantine with salicylates could decrease tinnitus‐like behaviors.

In a study conducted by Ricardo et al. on humans, with the similar methodology to our study, the efficacy of memantine in treating tinnitus was not found to be significant (Figueiredo et al. 2008). In their study, patients were administered memantine at the same dosage as in our study, but for a longer duration of 90 days, compared to our 60‐day treatment. This variation in results could be attributed to differences in treatment duration or even racial factors, underscoring the need for more comprehensive studies. Our study's findings are consistent with those of Yiwen et al., confirming memantine's effectiveness in reducing tinnitus severity (Zheng et al. 2012).

Our study has revealed that memantine is a promising option for treating tinnitus, a condition that affects millions of people worldwide. Our research has shown that memantine can effectively reduce the severity of tinnitus symptoms and related discomforts, leading to a marked improvement in the quality of life of individuals with tinnitus. Both the NRS and TSI measurement tools used in our study demonstrated the effectiveness of memantine in mitigating tinnitus symptoms.

Memantine is an NMDA receptor antagonist that regulates glutamate neurotransmission, which has been linked to the development of tinnitus. We believe that memantine's ability to modulate glutamate neurotransmission is what makes it an effective treatment for tinnitus. Although our findings suggest that memantine could be a viable treatment option for tinnitus, further investigations are necessary to confirm these results. Overall, our study provides valuable insights into the potential of memantine as a treatment option for tinnitus, and we hope that it will motivate further research in this area.

In our preliminary analysis, the presence of accompanying symptoms showed a significant association with higher final TSI scores. However, when included in the multivariate regression model alongside other clinical and demographic variables, this association was no longer statistically significant. This suggests that the apparent effect of comorbid symptoms may be confounded by other variables such as treatment group or occupational status. Future studies with larger samples may further clarify this relationship.

Multivariate analysis confirmed that the beneficial effect of memantine on tinnitus severity was robust and independent of other variables. The treatment group remained the sole significant predictor of final TSI scores.

Interestingly, factors, such as tinnitus duration, nature of tinnitus, or accompanying symptoms, did not show statistically significant effects in the model.

Throughout the course of our study, we encountered a number of limitations and challenges that warrant further examination. One of the most significant limitations was our failure to conduct a thorough evaluation of the side effects associated with memantine treatment. One limitation of this study is the use of the TSI as a primary outcome tool without prior published validation in the Iranian population. A major methodological limitation of this study was the use of cinnarizine as a baseline treatment in both groups. Although this approach was adopted to reflect empirical clinical practice in Iran and was based on the recommendations of the local Ethics Committee, it limited our ability to isolate the effect of memantine monotherapy. Moreover, although cinnarizine is frequently prescribed off‐label for tinnitus management, its efficacy remains unsupported by strong peer‐reviewed evidence. In our study, the placebo group—which received cinnarizine alone—did not demonstrate any meaningful reduction in tinnitus severity, suggesting that cinnarizine by itself may not offer therapeutic benefit. In contrast, the group that received both cinnarizine and memantine showed substantial improvement, implying that the observed clinical benefit is most likely attributable to memantine. Future studies may consider including a true placebo group without any concurrent pharmacological treatment to more precisely assess the independent effect of memantine.

As a result, further research is necessary to shed light on these issues. We recommend that future studies be conducted with larger samples and should focus on the long‐term efficacy of memantine, as well as its safety profile, potential side effects, and optimal dosing regimen, and that particular attention be paid to addressing the limitations and challenges encountered in our study. By doing so, we can hope to develop a more comprehensive understanding of the efficacy of memantine treatment for tinnitus and improve the quality of care for those suffering from this condition.

## Author Contributions

**Babak Pourayyoubi**: investigation, writing–original draft, writing–review and editing, data curation, methodology, project administration. **Alireza Rezaei‐Ashtiani**: supervision, conceptualization, writing–review and editing, project administration. **Javad Javaheri**: methodology, formal analysis, writing–review and editing, software. **Mohsen Ebrahimi Monfared**: conceptualization, writing–review and editing. **Farzad Zamani**: conceptualization, supervision.

## Peer Review

The peer review history for this article is available at https://publons.com/publon/10.1002/brb3.70697

## Data Availability

The data that support the findings of this study are available on request from the corresponding author. The data are not publicly available due to privacy or ethical restrictions.
